# Oral exposure to bisphenol S is associated with alterations in the oviduct proteome of an ovine model, with aggravated effects in overfed females

**DOI:** 10.1186/s12864-024-10510-z

**Published:** 2024-06-12

**Authors:** Coline Mahé, Marie-Emilie Lebachelier de la Riviere, Olivier Lasserre, Guillaume Tsikis, Daniel Tomas, Valérie Labas, Sébastien Elis, Marie Saint-Dizier

**Affiliations:** 1 INRAE, CNRS, Université de Tours, PRC, Nouzilly, 37380 France; 2 INRAE, PAO, Nouzilly, 37380 France; 3 INRAE, Université de Tours, CHU de Tours, Plateforme de Phénotypage Par Imagerie in/eX Vivo de L’ANImal À La Molécule (PIXANIM), Nouzilly, 37380 France

**Keywords:** Bisphenol S, Diet, Mass spectrometry, Oviduct, Secretions, Protein, Proteomic, Sheep, Ovine

## Abstract

**Background:**

Bisphenol S (BPS) is a substitute for bisphenol A in plastic manufacturing and, as a potential endocrine disruptor, may alter the physiology of the oviduct, in which fertilization and early embryo development take place in mammals. The objective of this study was to assess the effect of a daily dietary exposure to BPS combined with a contrasted diet on the oviduct fluid proteome using an ovine model.

**Results:**

Eighty adult cyclic ewes were allotted to four groups (20/group): overfed (OF) consuming 50 µg/kg/day of BPS in their diet, underfed (UF) consuming 50 µg/kg/day of BPS, and non-exposed controls in each diet group. After three months, the mean body condition score, plasma levels of glucose and non-esterified fatty acids were significantly higher in OF than in UF females. The proteins in collected OF samples (50 µg) were analyzed by nanoliquid chromatography coupled with tandem mass spectrometry (nanoLC-MS/MS). Overall, 1563 proteins were identified, among which 848 were quantified. Principal component analysis of the data revealed a clear discrimination of samples according to the diet and a segregation between BPS-exposed and non-exposed females in overfed ewes. Hierarchical clustering of differentially abundant proteins (DAPs) identified two clusters of 101 and 78 DAPs according to the diet. Pairwise comparisons between groups revealed a stronger effect of BPS in OF than in UF females (70 vs. 24 DAPs) and a stronger effect of the diet in BPS-exposed than non-exposed females (56 vs. 36 DAPs). Functional analysis of DAPs showed an enrichment in metabolic processes, immune system, cell response to stress, and reproductive processes.

**Conclusions:**

This work highlights for the first time the important impact of BPS on the oviduct proteome, with larger effects seen in OF than UF females. These results, together with previous ones, raise health concerns for everyone and call for a greater regulation of BPS in the food industry.

**Supplementary Information:**

The online version contains supplementary material available at 10.1186/s12864-024-10510-z.

## Background

The oviduct is the site of crucial reproductive events leading to pregnancy. This tubular organ and its fluid provide an optimal environment for sperm final migration and acquisition of fertilizing ability, fertilization and early embryo development [1]. The oviduct fluid originates from an ultrafiltration of the circulating plasma, de novo secretions from luminal oviduct epithelial cells and putative inputs from the follicular fluid at the time of ovulation, resulting in a complex mixture of proteins, metabolites, carbohydrates, lipids and hormones [2, 3]. Proteins are major components in the oviduct fluid and have been shown to play critical roles in sperm survival, gamete interaction, and embryo quality [4–8]. In addition, the proteomic composition of the oviduct fluid greatly varies according to the systemic and topical concentrations of estradiol and progesterone across the cycle in mammalian females [9–11]. Although poorly explored, there is growing evidence that the mammalian oviduct is also highly sensitive to environmental factors such as diet habits [12–14], heat stress [3, 15], or chemical compounds commonly found in our daily life [16, 17].

Bisphenols are plasticizers used worldwide in food and drinks packaging [18], but also in sale receipts [19] and laboratory and medical equipment [20, 21]. Food is considered to be the main route of exposure of adult and young people to bisphenols [22]. Due to its steroid hormone-like properties, bisphenol A (BPA) has been recognized as an endocrine disruptor and banned in food and drink containers in several industrialized countries (Canada, France, Belgium, Denmark, Australia, etc.…) [23]. Most commonly, BPA is replaced by other bisphenols such as BPS or BPF, having similar endocrine-disrupting properties as BPA [24]. Our study focus on BPS as it is one of the main substitutes of BPA in Europe and because our group previously reported that BPS was at higher levels in the follicular fluid of women compared to other bisphenols (BPF and BPAF) in France [25]. In addition, bisphenols are fat-soluble and may accumulate in fatty tissues [26]. Recent studies in pigs revealed that, after oral exposure to equal doses of BPS and BPA, the plasmatic concentration of BPS was 250 times higher than that of BPA [27], suggesting a higher body accumulation of BPS compared to BPA.

Sheep represent a good model to study the impact of endocrine disruptors in interaction with body mass index on human reproduction as it has already been used in previous toxicology studies [14, 28], and because of its similarities in the anatomy of the oviducts and kinetics of ovogenesis and folliculogenesis with women [29, 30].

In our previous study, oral exposure of adult ewes to BPS decreased estradiol concentration in the oviduct fluid in over- but not under-weight females [14], suggesting an aggravated effect of BPS in overfed animals. Therefore, the objective of the present study was to investigate the effect of daily oral exposure to BPS on the oviduct fluid proteome using an ovine model with contrasted diets.

## Results

**Fig. 1:**
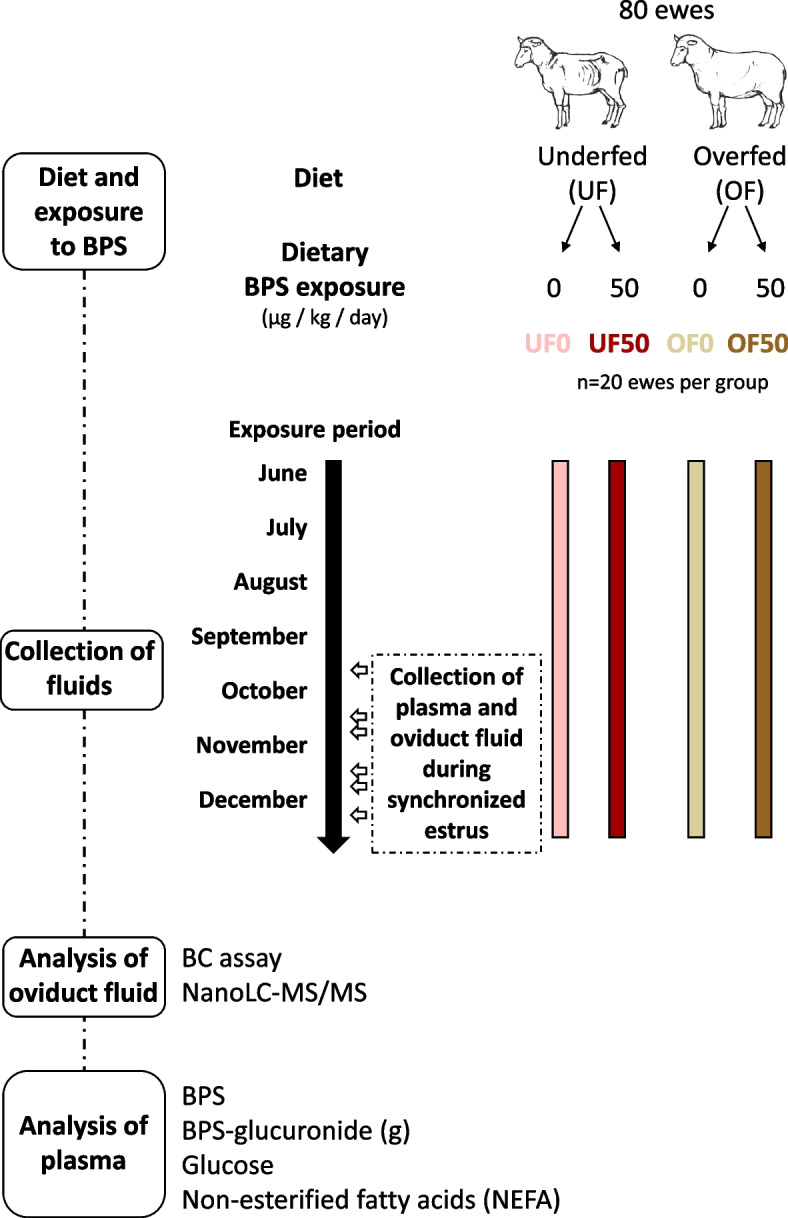
Experimental design (adapted from [14]). A total of 80 ewes were allocated to four groups: underfed diet without BPS exposure (UF0; *n* = 20), underfed diet with 50 µg/kg/day of BPS (UF50; *n* = 20), overfed diet without BPS exposure (OF0; *n* = 20), and overfed diet with 50 µg/kg/day of BPS (OF50; *n* = 20). Plasma and oviduct fluid were collected during the three months (between September and December) after synchronization of estrus, and ewes were slaughtered 2 days after eCG administration

The experimental design used for this study is presented in Fig. 1. The underfed (UF) and overfed (OF) ewes were divided into four experimental subgroups according to their dietary exposure to BPS: UF0 and OF0 as not exposed controls, and UF50 and OF50 exposed to 50 µg/kg/day of BPS. All ewes were at the pre-ovulatory stage of cycle at the time of oviduct fluid collection.

## Validation of the diet and BPS exposure models

The validation of the diet (OF vs. UF) and BPS exposure (0 vs. 50 µg/kg/day) models have been previously published [14]. At the time of oviduct fluid collection, significant differences in the mean body weight (BW; 55.7 ± 0.8 vs. 66.7 ± 0.8 kg; *p* < 0.001), body condition score (BCS; 2.2 ± 0.1 *vs.* 3.1 ± 0.1; *p* < 0.001), plasma glucose (3.7 ± 0.1 vs*.* 4.6 ± 0.2 mM; *p* < 0.001), and non-esterified acids (NEFA; 152.6 ± 12.7 vs*.* 216.9 ± 23.0 µM; *p* = 0.018) concentrations between OF and UF ewes were evidenced. Furthermore, while BPS and its metabolite BPS-g were undetectable in the plasma of unexposed females (UF0 and OF0 groups), both compounds were detected in the plasma of exposed ewes (UF50 and OF50 groups), with no difference between diets (BPS: 2.2 ± 0.6 vs. 2.0 ± 0.7 nM; BPS-g: 193.8 ± 20.1 vs. 197.0 ± 22.5 nM).

### Proteins identified in the ovine oviduct fluid and predicted secretory pathways

**Fig. 2:**
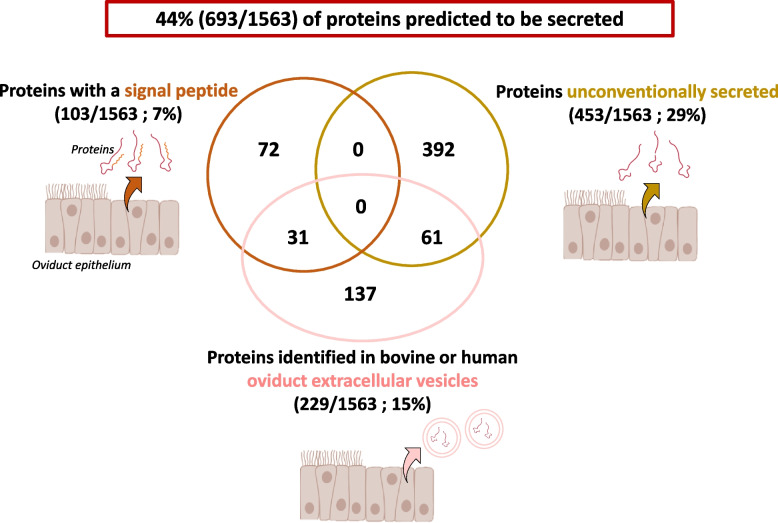
Distribution of proteins identified in the ovine oviduct fluid according to their secretion pathways. The number of proteins conventionally (with a signal peptide) and unconventionally secreted were predicted using Outcyte and Signal P online tools. The number of proteins identified in oviduct extracellular vesicles were assessed using previous proteomic data in bovine [31] and human [32] oviduct extracellular vesicles

A total of 1563 proteins were identified in the ovine oviduct fluid (see all proteins with their accession number, gene symbol, molecular weight, and normalized quantitative value in Table S1). Furthermore, 7% of identified proteins (103/1563) contained a signal peptide and were predicted to be classically secreted, while 29% of proteins (453/1563) were predicted to be secreted by unconventional pathways in the oviduct fluid (Fig. 2 and Table S1). In addition, 15% of proteins (229/1563) were previously reported in oviduct extracellular vesicles (EVs) from the bovine [31] or human [32] species (Fig. 2 and Table S1). Overall, 44% of proteins (693/1563) were predicted to be secreted in the oviduct fluid.

### Profiling of the oviduct fluid proteome according to the exposure to BPS and diet

**Fig. 3:**
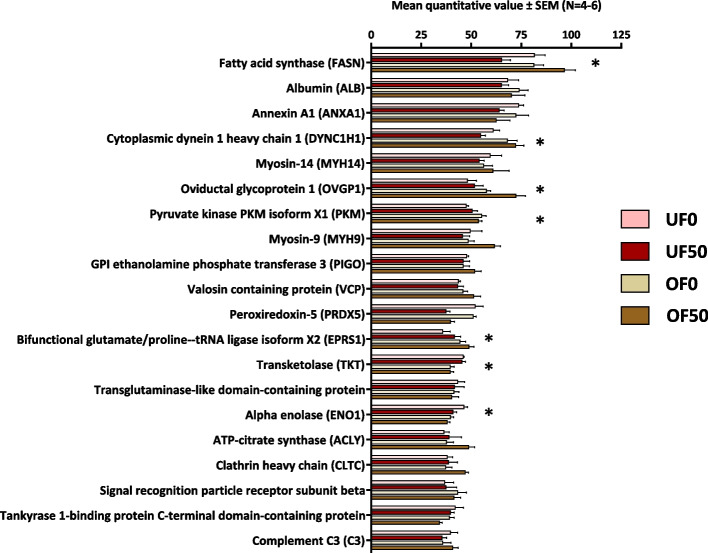
Mean quantitative value of the 20 most abundant proteins in all samples. The mean quantitative value corresponds to the mean normalized weighted spectra (NWS) of each protein in each condition. UF0 = underfed ewes non-exposed to bisphenol S; UF50 = underfed ewes exposed to 50 µg/kg/day of bisphenol S; OF0 = overfed ewes non-exposed to bisphenol S; OF50 = overfed ewes exposed to 50 µg/kg/day of bisphenol S; * = differentially abundant proteins after global ANOVA

A total of 848 proteins were retained for quantitative analysis, i.e. proteins with a mean quantitative value of at least 2 in at least one condition (Table S1). Overall, the 20 most abundant proteins included fatty acid synthase (FASN), annexin A1 (ANXA1), oviductin (OVPG1), myosins (MYH9 and MYH14), peroxiredoxins (PRDX2 and PRDX5), transketolase (TKT), alpha enolase (ENO1), and complement C3 (C3) (Fig. 3).

**Fig. 4:**
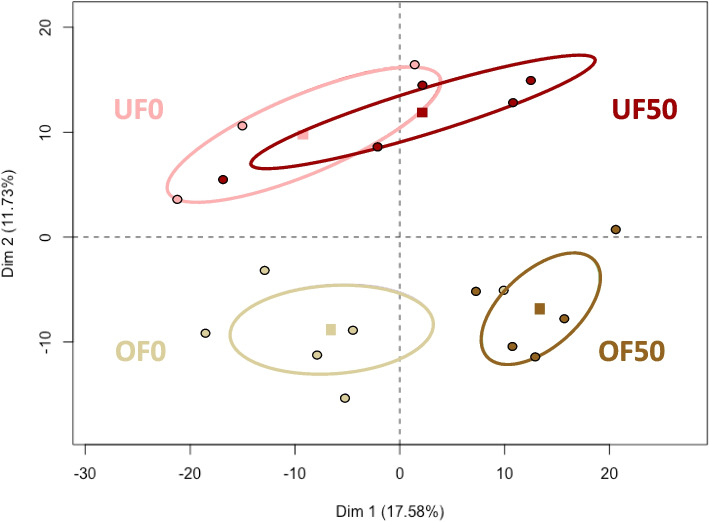
Principal component analysis of the 848 quantified proteins in under- and overfed ewes exposed or not to 50 µg/kg/day of bisphenol S. One dot represents one oviduct fluid sample. Squares represent the mean of data, and ellipses represent the 95 % interval of confidence for each group. The percentage on each axis (dimension) represents the total variance of data according to the diet (horizontal axis) and exposure to BPS (vertical axis). UF0 = underfed ewes non exposed to bisphenol S; UF50 = underfed ewes exposed to 50 µg/kg/day of bisphenol S; OF0 = overfed ewes non exposed to bisphenol S; OF50 = overfed ewes exposed to50 µg/kg/day of bisphenol S

The principal component analysis (PCA) of all quantified proteins revealed a clear separation of the samples according to the diet (under vs*.* overfed; vertical axis in Fig. 4). Furthermore, in the OF group of ewes, the samples segregated according to the exposure to BPS (OF0 vs. OF50; horizontal axis in Fig. 4).

**Fig. 5:**
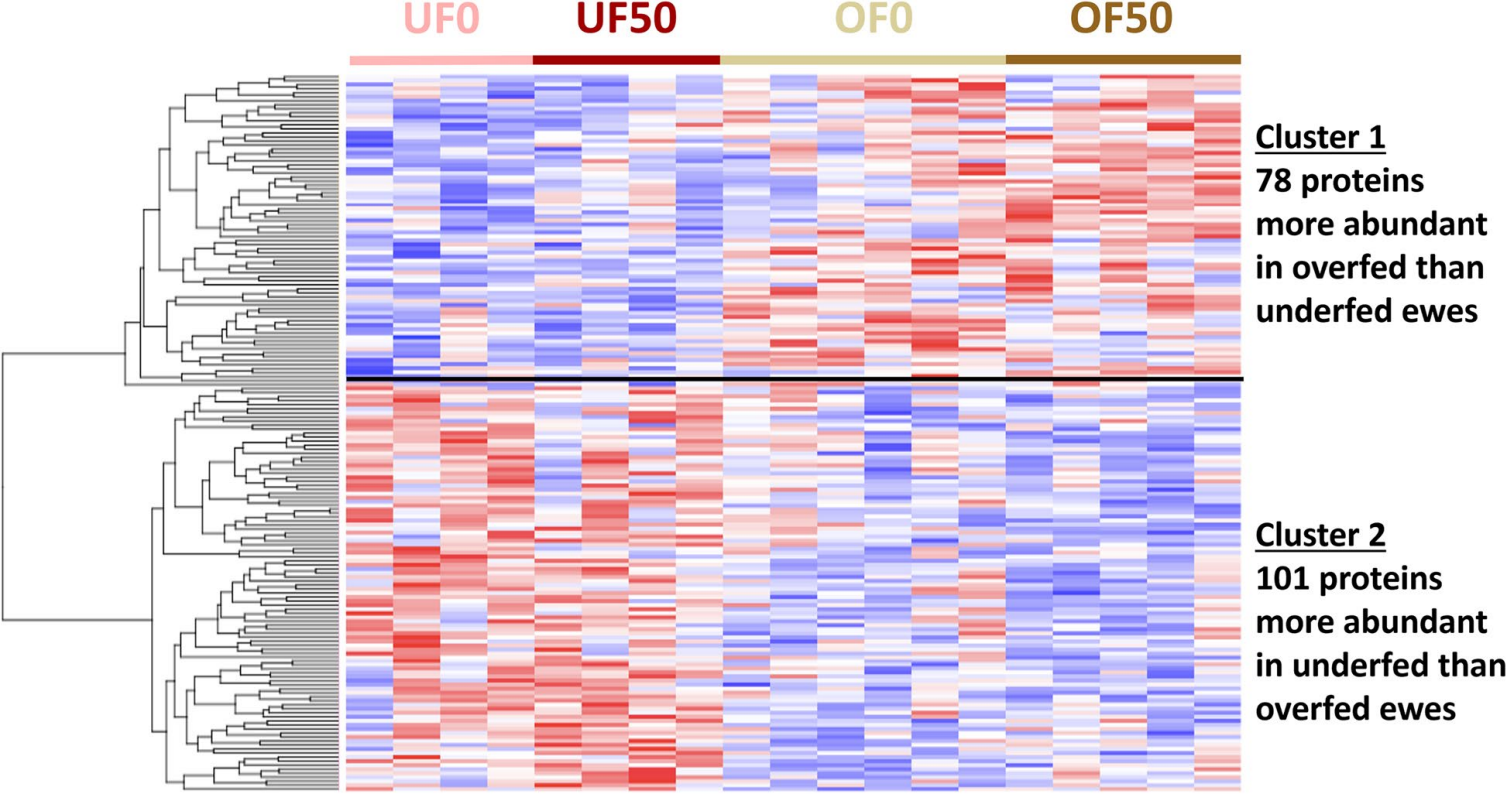
Heatmap representation of the hierarchical clustering of the 179 differentially abundant proteins (**A**) and functional enrichment analysis of the two proteins clusters (**B**). **A** Heatmap of the 179 differentially abundant proteins after global analysis of variance (*p*-value ≤ 0.05). Each line corresponds to the mean quantitative value in one sample. Each column represents one protein: blue means lower abundance and red means higher abundance compared to the other conditions. **B** Functional enrichment analysis of the two clusters from Metascape for Gene Ontology Biological Process and Reactome pathways. UF0 = underfed ewes non exposed to bisphenol S; UF50 = underfed ewes exposed to 50 µg/kg/day of bisphenol S; OF0 = overfed ewes non exposed to bisphenol S; OF50 = overfed ewes exposed to 50 µg/kg/day of bisphenol S

To more specifically evaluate which factors affected the most oviduct fluid proteome, a hierarchical clustering of the 179 differentially abundant proteins (DAPs) was performed (ANOVA *p*-value ≤ 0.05). The heat map representation of this analysis confirmed that the diet had a strong effect on the oviduct fluid proteome, and evidenced two clusters of DAPs: cluster 1 of 78 DAPs more abundant in OF than UF ewes, including proteins like FASN and OVGP1; and cluster 2 of 101 DAPs more abundant in UF than OF ewes, including proteins like ENO1 and TKT (see Fig. 5 and Table S2 for the complete list of DAPs).

The GO analysis of proteins in cluster 1 evidenced an enrichment in 16 BP and pathways, including two related to intracellular trafficking, five to metabolism, three to immune system, and one related to the negative regulation of reproductive process (Table 1). Analysis of proteins in cluster 2 showed an enrichment in 18 biological processes (BP) and pathways, including six related to metabolism, two related to the immune system, three to the cell response to stress, and three to cell movement among the most significant ones (*p*-value < 0.01) (Table 2).

**Table 1:** Metascape enrichment analysis of the differentially abundant proteins more abundant in the oviduct fluid of overfed than underfed ewes (cluster 1)

Category	Description	*Gene symbols*	-Log (*p*-value)
**Trafficking**	Intra-Golgi and retrograde Golgi-to- ER traffic	*DCTN1, DYNC1H1 ,KIF5B, RAB1A, COG1, TUBA1B, * *KIF13B, DYNC1LI1, KIF21A, COG3, TUBA1C, KLC3, * *MYO6, RAC1, SEC24C, G6PD, HSPA6, PSMB2, PSM1,* *RPS4X, RPS20, TXN, UBE2D3, ATP6V1F, PRDX6, * *GNPNAT1, CARMIL1, CDC37, PDLIM5*	-13.8
	Intracellular protein transport	*KIF5B, MYO6, RAB1A, SGTA, VPS26A, * *SEC24C, CDC37, KIF13B, COG3, * *DCTN1, CORO1A, DYNC1H1, CLUH*	-4.7
**Metabolism**	Metabolism of amino acids and derivatives	*CTH, EPRS1, GAMT, KARS1, PSMB2, PSME1, RPS4X, * *RPS20, RIDA, DYNC1H1, RAC1, SEC24C, TUBA1B, * *PYCARD, DYNC1LI1, TUBA1C, G6PD, TXN,PRDX6, * *YBX1, RTCB, WDR77, MUC1, DCTN1, KIF5B, CDC37, * *UBE2D3, COMMD9, VPS26A*	-7.0
	Selenoamino acid metabolism	*CTH, EPRS1, KARS1, RPS4X, * *RPS20, AARS1, RTCB*	-5.2
	Amino acid metabolic process	*AARS1, CTH, EPRS1, * *GART, KARS1, RIDA*	-4.5
	Regulation of mRNA metabolic process	*YBX1, YBX3, PRDX6, * *RIDA, WDR77*	-3.0
	Monocarboxylic acid metabolic process	*DBI, FASN, GAMT, * *IFT122, IFT140, PKM, ACOT11*	-2.3
**Immune system**	Neutrophil degranulation	*DYNC1H1, HSPA6, PKM, RAC1, * *PRDX6, VAT1, COMMD9, PYCARD, DYNC1LI1*	-6.1
	Cellular response to cytokine stimulus	*CTH, EPRS1, FASN, KIF5B, YBX1, * *YBX3, TUBA1B, CORO1A, CACYBP, PYCARD*	-5.5
	Phagocytosis	*MFGE8, RAC1, CORO1A, DCTN1, * *RAB1A, SGTA, SEC24C, ATP6V1F*	-2.3
**Reproduction**	Negative regulation of reproductive process	*OVGP1, YBX3, WDR77, MFGE8*	-3.2
**Other**	Positive regulation of cytoplasmic translation	*YBX1, PKM, YBX3, AARS1, * *EPRS1, RPS4X, RIDA*	-5.4
	Positive regulation of protein polymerization	*DCTN1, RAC1, PYCARD, CARMIL1, * *DYNC1H1, CORO1A, MFGE8, * *MYO6, RAB1A, TXN, PDLIM5, KARS1*	-4.3
	Signal transduction by p53 class mediator	*MUC1, MYO6, PYCARD, * *YBX3, CTH*	-3.0
	Endomembrane system organization	*DCTN1, RAB1A, SGTA, ATP6V1F, * *COG1, COG3, SEC24C*	-2.9
	Carboxylic acid biosynthetic process	*CTH, FASN, GAMT, * *PYCARD, G6PD, YBX1*	-2.5

**Table 2:** Metascape enrichment analysis of the differentially abundant proteins more abundant in the oviduct fluid of underfed than overfed ewes (cluster 2)

Category	Term description	*Gene symbols*	-Log (*p*-value)
**Metabolism**	Nucleobase-containing small molecule metabolic process	*ADSS2, ALDOA, ASMTL, ATP6V1A, GPD1L, HINT1, ISYNA1, MOCS3, MPI, MTAP, * *PGD, QNG1, TALDO1, TKT, UAP1L1*	-7.9
	Metabolism of carbohydrates	*ALDOA, ACAT2, ENO1, GOT1, PGD, PGK1, PGM1, RANBP2, * *TALDO1, TKT, TKFC, MPI, CBR1, GSTM1, MTAP, * *PHGDH, ISYNA1, QNG1, GPD1L*	-7.6
	Metabolism of amino acids and derivatives	*AHCY, ALDH7A1 ,GOT1, GSR, MTAP, PSMD3, RPL3, * *RPLP0, EEF1E1, PHGDH, PAK2, EIF2B5, NCL, RANBP2, * *EDC4, TP53RK, ARPC2,GIT1*	-6.7
	Glyceraldehyde-3-phosphate metabolic process	*TALDO1, TKT, TKFC, ATP6V1A, BLVRA, CRYAB, * *GSR, PGD, PSMD3, RANBP2, RPL3, RPLP0, DNAJA2, * *CCAR2, ALDH7A1 ,GPD1L, ACO1, PHGDH, PLCB3*	-6.3
	Aspartate family amino acid metabolic process	*ADSS2, GOT1,MTAP, PHGDH, AHCY, * *FARSA, YARS1, CBR1, GSTM1, GLRX3*	-4.9
	Polyol metabolic process	*GOT1, TKFC, ISYNA1*	-2.5
**Immune system**	Gene and protein expression by JAK-STAT signaling after Interleukin-12 stimulation	*MTAP, PAK2, RPLP0, TALDO1, PSMD3, * *PTPN13, USP14, PTPN23, RANBP2*	-5.3
	Interleukin-1 family signaling	*PSMD3, PTPN13, USP14, PTPN23*	-2.9
**Cell movement**	Microtubule-based movement	*MAP1B, RABL2B, DPCD, LZTFL1, IFT122, * *ROPN1L, TTC21A, RSPH9, PTPN23, RSPH1*	-4.7
	Cilium movement involved in cell motility	*DPCD, LZTFL1, ROPN1L, * *TTC21A, RSPH9*	-4.4
	Microtubule bundle formation	*MAP1B, RSPH1, RSPH9*	-2.2
**Response to stress**	Cell redox homeostasis	*GSR, GLRX3, GIT1, ACO1, * *ATP6V1A, ALDOA, CA2*	-3.6
	Cellular response to heat stress	*CRYAB, RANBP2, CCAR2, * *ENO1, GOLGA4, MAP1B*	-2.6
	Response to toxic substance	*GSR, GSTM1, MAP1B, PTPN13*	-2.4
**Other**	Regulation of supramolecular fiber organization	*CRYAB, MAP1B, PAK2, RNH1, * *WDR1, ARPC2, EML2, GIT1*	-4.7
	Cytosolic tRNA aminoacylation	*FARSA, YARS1, EEF1E1, * *RPL3, RPLP0, EIF2B5*	-4.3
	tRNA metabolic process	*FARSA, YARS1, MOCS3, * *QNG1, TP53RK*	-3.5
	Negative regulation of proteolysis	*CRYAB, PAK2, SERPINB5, USP14, * *CCAR2, ANXA8, ENO1, HECTD1*	-3.4

### Pair-wise comparisons between female groups: effect of exposure to BPS

**Fig. 6:**
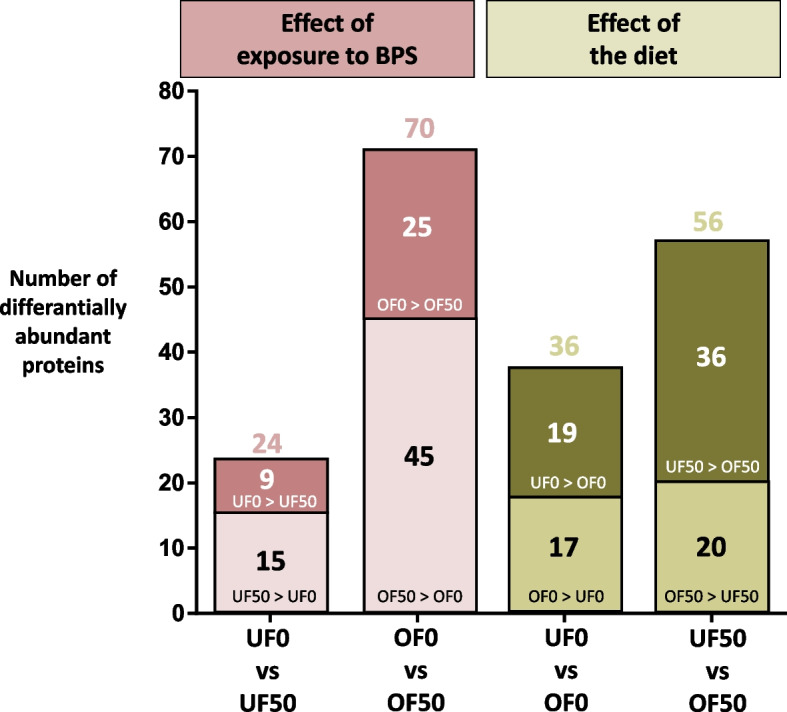
Number of differentially abundant proteins according to the bisphenol S exposure and the diet. Proteins were considered as differentially abundant using a Student’s t-test *p*-value ≤ 0.05 and a fold-change ratio ≥ 1.5. The number of over-abundant proteins are indicated inside the bar, and the total number of differentially abundant proteins per comparison are indicated on the top of the bars

In accordance with the results above, exposure to BPS induced a higher proportion of DAPs (t-tests *p*-value ≤ 0.05 and fold change ratio ≥ 1.5; Fig. 6 and Table S3 for the complete lists of DAPs in each comparison) in OF than UF ewes: 70 (8%) vs. 24 (3%) DAPs in the OF0 vs. OF50, and UF0 vs. UF50 pair-wise comparisons, respectively, with no difference between mean fold-change ratios (2.1 ± 0.1 vs. 1.8 ± 0.1, respectively). The GO analysis of the 70 DAPs after BPS exposure in OF ewes (OF0 vs. OF50) showed a significant enrichment in 21 biological processes and pathways, including six related to trafficking (vesicular transport, endocytosis…), four to metabolism (metabolism of carbohydrates, glutathione metabolism…), two to epithelium and cilia (cilium assembly), and four to cellular response to DNA damage stimulus (Table 3). The DAPs after BPS exposure in UF ewes (UF0 vs. UF50) were over-represented in five BP GO terms and pathways, including the immune system (neutrophil extracellular trap formation) and reproduction (placenta development; Table 4).

**Table 3:** DAVID enrichment analysis of the differentially abundant proteins in the oviduct fluid of BPS-exposed vs. control ewes in the overfed group (OF0 vs. OF50)

Category	Term name	*Gene symbols*	*P*-value
**Trafficking**	Intracellular protein transport	*ARF4, MYO6, STXBP2, RAB3GAP2, AP2B1, SEC24C, IPO4*	0,0003
	Membrane trafficking	*HSPA8, ARF4, YWHAQ, ARPC2, MYO6, EXOC4, RAB3GAP2, AP2B1,SEC24C, RAB7A*	0,001
	Vesicle-mediated transport	*HSPA8, ARF4, YWHAQ, ARPC2, MYO6, EXOC4, RAB3GAP2, AP2B1, SEC24C, RAB7A*	0,001
	Endocytosis	*HSPA8, ARFGEF2, ARF4, ARPC2, AP2B1, RAB7A*	0,004
	Intraciliary retrograde transport	*IFT140, IFT122*	0,039
	Exocytosis	*ARFGEF2, EXOC4, LLGL2*	0,042
**Metabolism**	Glutathione metabolic process	*GSTM3, GPX1, OPLAH*	0,008
	Glutathione metabolism	*GSTM3, GPX1, OPLAH*	0,024
	Glycogen catabolic process	*AGL, PFKM*	0,028
	Metabolism of carbohydrates	*SHPK, AGL, SORD, GAPDH, PFKM*	0,033
**Cilia**	Protein localization to cilium	*ARF4, IFT140, IFT122*	0,005
	Cilium assembly	*ARF4, IFT140, EXOC4, IFT122*	0,027
**Response to stress**	Cellular response to DNA damage stimulus	*STK11, PAXX, UBA6, CCAR2*	0,050
**Other**	Salmonella infection	*CYFIP1, ARPC2, MYO6, EXOC4, GAPDH, RAB7A*	0,004
	Pathogenic Escherichia coli infection	*CYFIP1, ARPC2, MYO6, SEC24C, GAPDH*	0,009
	Dendrite extension	*CYFIP1, STK11*	0,028
	Mitochondrial fragmentation involved in apoptotic process	*FIS1, CCAR2*	0,028
	Regulation of protein stability	*HSPA8, USP4, CCAR2*	0,030
	Positive regulation of mRNA catabolic process	*MOV10, UPF1*	0,039
	Dendritic spine development	*ARF4, UBA6*	0,039
	3'-UTR-mediated mRNA destabilization	*MOV10, UPF1*	0,047

**Table 4:** DAVID enrichment analysis of the differentially abundant proteins in the oviduct fluid of BPS-exposed vs. control ewes in the underfed group (UF0 vs. UF50)

Category	Term name	*Gene symbols*	*P*-value
**Immune system**	Neutrophil extracellular trap formation	*FGA, PLCB3, HDAC6*	0,013
**Trafficking**	Intra-Golgi and retrograde Golgi-to-ER traffic	*GOLGA4, DCTN1, IGF2R*	0,023
**Reproduction**	Placenta development	*CUL7, RTCB*	0,038
**Other**	Diseases of signal transduction by growth factor receptors and second messengers	*FGA, GOLGA4, DCTN1, HDAC6*	0,013
	Positive regulation of dendrite morphogenesis	*CUL7, HDAC6*	0,023

### Pair-wise comparisons between female groups: effect of the diet

The overfeeding induced higher proportions of DAPs in ewes exposed to BPS compared to the non-exposed controls: 56 (7%) vs. 36 (4%) DAPs in the UF50 vs. OF50 and UF0 vs. OF0 pair-wise comparisons, respectively, with no difference between mean fold-change ratios (2.0 ± 0.1 vs. 2.1 ± 0.1, respectively). The GO analysis of the DAPs between UF50 and OF50 showed a significant enrichment in four BP GO terms and pathways, including two in metabolism (metabolic and glucagon signaling pathway); one in reproduction (flagellated sperm motility) and one in the morphogenesis of an epithelium (Table 5). The DAPs between UF0 and OF0 were overrepresented in two GO terms including lipid metabolic process and localization (Table 6).

**Table 5:** DAVID enrichment analysis of the differentially abundant proteins in the oviduct fluid of underfed vs. overfed ewes in the BPS-exposed group (UF50 vs. OF50)

Category	Term name	*Gene symbols*	*P*-value
**Metabolism**	Metabolic pathways	*GSTM3, PLCB3, CA2, GSR, UAP1L1, PYCR3, GART, PFKM, DERA*	0,019
	Glucagon signaling pathway	*PLCB3, PRMT1, PFKM*	0,026
**Reproduction**	Flagellated sperm motility	*TTC21A, DPCD, LZTFL1*	0,020
**Epithelium**	Morphogenesis of an epithelium	*CA2, SERPINB5*	0,048

**Table 6:** DAVID enrichment analysis of the differentially abundant proteins in the oviduct fluid of underfed vs. overfed ewes in the control group (UF0 vs. OF0)

Category	Term name	*Gene symbols*	*P*-value
Metabolism	Lipid metabolic process	*RIDA, CTH, ACAT2*	0,037
Other	Localization	*MUC1, RAC1*	0,05

## Discussion

To our knowledge, this is the first study evaluating the effect of a long-term oral exposure to BPS on the proteome of the oviduct, an organ central for early reproductive events in vivo. A total of 1563 proteins were identified, which represented the most exhaustive list in the ovine oviduct fluid so far, as only 624 proteins were identified in a previous proteomic study in the same species [11]. The main outcomes of this study are that 1) a dietary exposure to 50 µg/kg/day BPS altered protein abundance with more significant effects in overweighted than underweighted ewes, in line with our initial hypothesis, 2) the diet had a strong impact on the oviduct proteome, which was slightly higher in BPS-exposed ewes than non-exposed controls and, 3) proteins altered by BPS exposure were mostly involved in cell metabolism, response to stress and immune system. Following the same experimental design, a previous study from our lab identified changes in the concentration of estradiol in the ovine oviduct secretions [14], supporting the fact that BPS operate changes in the milieu in which fertilization and early embryo development take place.

### Origins and secretory pathways of proteins identified in the oviduct fluid

From the list of identified proteins, 44% were predicted to be secreted including 7% with a signal peptide and 29% unconventionally secreted. These proportions are similar to those previously reported in a previous study in the bovine oviduct fluid [33]. The remaining 56% of proteins may originate from other pathways of secretion, apocrine or non-canonical [34] and/or the significant renewal of the oviduct epithelium during estrus [35]. The oviduct fluid is constituted of a complex mixture of proteins from various origins which may have or not a role on oviduct morphology, sperm fertilizing ability, fertilization and embryo development. The oviduct fluid at the pre-ovulatory stage probably contains proteins that 1) have already fulfilled their role in the oviduct during previous cycles, 2) are currently involved in gametes and embryo development, and 3) can be involved later. In our study, the temporal involvement of proteins is difficult to distinguish, which is one of our limitations.

### Impact of exposure to BPS on the oviduct proteome and functional implications

The PCA and the number of DAPs between exposed and non-exposed ewes to BPS revealed that OF ewes were more sensitive to BPS than UF ewes. In agreement, a previous study pointed out a contrasted effect of BPS on the steroid hormone composition of the oviduct fluid according to the diet [14]. Indeed, OF ewes exposed orally to 50 µg/kg/day BPS had a significantly lower concentration of estradiol in the oviduct fluid compared to non-exposed ewes, but this significant BPS effect on estradiol was not found in UF ewes [14]. In humans, a study conducted in the USA showed a higher concentration of BPS in the urine of obese compared to non-obese male and females adults [36], suggesting that the lifestyle and diet likely influence the bioavailability of BPS in the body.

In the oviduct, spermatozoa use glucose as a source of energy for their acquisition of fertilizing ability [37]. After fertilization, the embryo first uses lactate and pyruvate then progressively switches to glucose for its metabolism [38]. In the OF ewes, exposure to BPS had a particularly strong impact on enzymes taking part in the metabolism of glucose, including glyceraldehyde-3-phosphate dehydrogenase (GAPDH; OF0:OF50 ratio = 1.69 and t-test *p*-value = 0.02), ATP-dependent 6-phosphofructokinase (PFKM; OF50:OF0 ratio = 3.1 and t-test *p*-value = 0.017). and glycogen debranching enzyme (AGL; OF50:OF0 ratio = 1.51 and t-test *p*-value = 0.003). In agreement, in mice, a 10-week oral administration of 50 µg/kg/day BPS through water increased the gene expression of glucose-6-phosphatase (G6Pase) and phosphoenolpyruvate carboxykinase (PEPCK), two proteins also involved in carbohydrate metabolism, in the intestinal epithelium and liver [39]. The in vivo induction of hyperglycemia or the in vitro production of embryo with high concentration of glucose in the culture medium both increased the proportion of apoptotic cells in blastocysts in mice [40].

The mammalian epithelium lining the oviduct is composed of secretory and ciliated cells in which cilia are essential for sperm binding in the reservoir but also for the transport of gametes and embryo [38]. Along with metabolic process, BPS in OF ewes changed the abundance of proteins involved in cilium assembly. In particular, the intraflagellar transport proteins IFT122 (OF50:OF0 ratio = 1.86 and t-test *p*-value = 0.02) and IFT140 (OF50:OF0 ratio = 1.89 and t-test *p*-value = 0.012) were more abundant in the oviduct fluid of exposed than non-exposed ewes. In mice, IFT122 KO in the retinal epithelium impaired cilia morphogenesis and protein trafficking [41]. This result suggests that BPS may alter the ciliary function of the oviduct epithelium.

The maintenance of the balance between reactive oxygen species (ROS) and antioxidant enzymes in the oviduct is crucial for oocyte maturation, sperm capacitation and embryo development [42]. In the oviduct fluid of OF ewes, BPS modified the abundance of proteins involved in the cellular response to oxidative stress. In particular, glutathione S-transferase Mu 3 (GSTM3; OF0:OF50 ratio = 1.96 and t-test *p*-value = 0.006) and gluthanione peroxidase (GPX1; OF0:OF50 ratio = 1.63 and t-test *p*-value = 0.008), two enzymes involved in the antioxidant activity of cells, were found to be less abundant in exposed than non-exposed ewes. In agreement, a subcutaneous administration of 50 µg/kg/day BPS in mice decreased the concentration of antioxidant enzymes in the ovarian tissue such as glutathione peroxidase (GPX) and catalase (CAT) [43]. These results suggest that BPS decreased the availability of antioxidants in the oviduct of exposed ewes. In mice, a 21-day intraperitoneal administration of 50 µg/kg/day BPS increased the level of lipid peroxidation in oocytes, which further decreased fertilization rate and the proportion of 2-cell embryos and blastocysts compared to non-exposed control females [44]. Accordingly, after in vitro maturation of oocytes with 10 nM BPS, embryos had a lower blastocyst rate compared to maturation without BPS in sheep [45]. In pigs, after 3 h of co-incubation, BPS at 1 and 100 µM increased sperm mitochondrial ROS, which further decreased their progressive motility [46]. Thus, it is probable that long-term exposure to BPS decreased antioxidant enzymes in the oviduct fluid which may lead to alterations in fertilization and early embryo development.

In this study, we did not investigate the effect of BPS on female fertility but we analyzed its effect as an estrogen mimetic on the oviduct proteome at the pre-ovulatory stage as the level of estrogen at this stage in the oviduct fluid is higher than at other stages [10]. In addition, the impact of BPS on the oviduct fluid proteome was studied in absence of artificial insemination in order to evaluate in parallel the effect of BPS on the pre-ovulatory follicle and oocyte [14] and to avoid any inputs of sperm and embryos on the oviduct proteome.

### Interaction between diet and exposure to BPS

We observed that the diet had a different impact on the OF proteome according to whether or not they were exposed to BPS. Indeed, the exposure to BPS enabled us to identify more than twofold more DAPs in OF ewes compared to UF ewes. The oviduct fluid from OF ewes could therefore be more sensitive to BPS, leading to a potential impact on fertilization and embryo development.

After following a different diet for three to six months, we observed in the plasma of ewes an increased concentration of NEFA of OF compared to UF ewes. In cattle, oviduct epithelial cells cultured with elevated apical concentrations of NEFA decreased the transepithelial electric resistance and increased the level of lipid droplets in cells [47]. In addition, in the same species, a co-culture of embryos with oviduct epithelial cells pre-exposed to high levels of NEFA decreased cleavage rate compared to a control co-culture with cells non-exposed to NEFA [48]. Therefore, the greater effect of BPS on the oviduct proteome may also be due to the increase in plasma NEFA in OF ewes.

### Impact of the diet on the oviduct proteome and functional implications

The PCA showed a clear separation between oviduct fluids from UF vs. OF ewes, suggesting the significant impact of the diet in regulating protein abundance in the ovine oviduct fluid. This is in agreement with previous transcriptomic and proteomic analysis of oviduct epithelial cells demonstrating differentially abundant genes and proteins between a control and OF diet in sheep [13] and mice [12]. In addition, a restricted or high fat diet during 15 days was shown to alter the abundance of the estrogen-dependent glycoprotein 1,2 and 3 in the oviduct fluid of peripubertal sows [49]. However, a previous study from our lab using the same experimental design demonstrated that the concentration of estrogens in the same oviduct fluids did not change between underfed and overfed ewes, suggesting an estrogen-independent effect of diet on the oviduct proteome [14].

Regardless of exposition to BPS, DAPs more abundant in the oviduct fluid of UF than OF ewes (cluster 2 of the heatmap in Fig. 5) were specifically involved in cell movement including microtubule-based movement, cilium movement involved in cell motility and microtubule bundle formation. Ciliary beating from the ciliated cells of the oviduct epithelium induced a fluid flow, which is crucial for transporting the oocyte and embryo, and for spermatozoa, that are able to move against the flow in a mechanism called rheotaxis [38]. The disturbance of cilia motility could make pregnancy difficult to achieve as it was described in several cases in women suffering from Kartagener syndrome, i.e. presenting immotile cilia in the respiratory and oviductal epithelium [50]. The results of the present study thus suggest that cilia development and function may be impaired by the diet habits, therefore aggravating potential fertility issues.

Moreover, the proteins more abundant in UF than OF ewes were also involved in cell redox homeostasis, like the glutathione family, the glutathione reductase (GSR), the glutharedoxin-3 (GLRX3) and the glutathione S-transferase Mu 1 (GSTM1). In goats, the levels of glutathione reductase and transferase, as well as the antioxidant activities in the circulating plasma were also increased after an under- compared to an overfed diet [51]. In addition, we showed that two other proteins from the glutathione family, GPX1 and GSTM3, were less abundant in the oviduct fluid of ewes exposed to BPS compared to non-exposed ones. Therefore, the diet and BPS both had an impact on antioxidant enzymes, disrupting the redox status in the oviduct. Future investigations will more specifically evaluate the effect of the diet and BPS on the production of reactive oxygen species in the oviduct lumen and the potential impact on gametes and embryo development.

The DAPs more abundant in the oviduct fluid of OF than UF ewes (cluster 1 of the heatmap in Fig. 5) were involved in the regulation of reproductive processes. In another study, female goats fed 1.3, 1.6 and 1.9 times their nutritional requirements evidenced seven DAPs, including the alpha enolase (ENO1), which is involved in process related to fertilization and early embryo development [13].

## Conclusions

For the first time, this in vivo study shows that a long-term oral exposure to BPS altered the protein content of the oviduct of ewes, with a higher impact in heavier females. Various functional pathways with potential impact on early reproductive events were affected. These results, together with previous ones on the deleterious effects of BPS and other bisphenols on the reproductive system, raise health concerns for everyone and call for a greater regulation of not only bisphenol A, but all bisphenols, in the food industry.

## Methods

### Animals and experimental design

The experimental design is presented in Fig. 1 and was carried out over two years in the INRAE Experimental Unit of Animal Physiology of the Orfrasière (PAO, Nouzilly, France) as previously described [14]. In June, i.e. three months before sample collection, a total of 80 ewes (primiparous non-pregnant Ile-de-France owned by INRAE Experimental Unit PAO) with similar age 5 (2.55 ± 0.04 in average), BW and BCS were allotted into two diet groups: one UF group (*n* = 40) receiving 50% of their daily food requirements (DFR) (0.15 kg of feed/animal) until a median BCS of 2 was reached then 80% of DFR (0.24 kg of feed/animal) and one OF group (*n* = 40) receiving 165% of their DFR (0.5 kg of feed/animal) to reach a median BCS of 4. Briefly, the diet consisted in varying the quantity of a wheat-based food supplement (Agneau-echange, AXEREAL Elevage, Saint-Germain-de-Salles, France). From the start of the experiment, the UF and OF groups were divided into four experimental subgroups according to their dietary exposure to BPS: UF0 and OF0 as not exposed controls, and UF50 and OF50 exposed to 50 µg/kg/day of BPS. The BW and BCS of all females were monitored once a month up to the time of slaughter.

Between September and December, i.e. during the natural breeding season, all ewes were synchronized for estrus in batches of 6–8 ewes (including 1–2 ewes per experimental group) with a vaginal progesterone sponge (Chrono-Gest® 20 mg, MSD, Beaucouze, France) for 11 days, followed by an intramuscular administration of eCG (Synchro-Part® PMSG 400 UI, CEVA Santé Animale, Libourne, France) to induce final follicular growth. Two days after eCG administration, i.e. at the presumptive day of ovulation [52], ewes were rendered unconscious by electronarcosis then bled in the INRAE experimental slaughterhouse (Nouzilly, France). Blood samples (5 mL) were collected at the time of slaughterhouse bleeding in heparinized tubes (17 IU/mL sodium heparin, Vacutainer®; Becton Dickinson and Company, Le Pont de Claix, France), centrifuged (3,700 g for 30 min at 4 °C) and plasma samples were stored at -20 °C for further assay of glucose, NEFA, BPS and its metabolite BPS-glucuronide (BPS-g).

### Collection and preparation of oviduct fluids

Oviduct fluids were collected within 15 min after death, as previously described [14]. Briefly, pairs of ovaries and oviducts from individual ewes were placed on ice and after elimination of the surrounding tissue and infundibulum, the oviduct fluid was collected from the isthmus to the ampulla by gentle squeezing with a sterile glass slide. Only the oviduct fluid ipsilateral to the preovulatory follicle were collected for proteomic analysis. In case of bilateral ovulations, both oviducts were collected. The oviduct fluids were isolated from cells by centrifugation (12,000 g for 15 min, 4 °C), their volumes were recorded by pipetting and all samples were stored at -80 °C until further analysis.

Preliminary experiments showed that 50 µL was the minimal volume required for the analysis by chromatography and mass spectrometry of all steroid hormones [14] and proteins in the oviduct fluid. On the basis of the average volume collected per oviduct (10–15 µL), pools of 4–7 ewes were made to reach a final volume of 50 to 60 µL (from 52 to 68 µL). The contribution of individual ewes accounted for a maximum of 50% of the final volume of pools. Totals of 4–6 biological replicates (pools of ewes) per experimental group were constituted (UF0, *n* = 4; UF50, *n* = 4; OF0, *n* = 6; OF50, *n* = 5). In the following, the term “sample” refers to these pools of oviduct fluid. Samples were assayed for protein concentration (Uptima BC Assay kit; Interchim, Montluçon, France) according to the manufacturer’s instructions and using bovine serum albumin as standard. The mean protein concentration in the oviduct fluid samples was 67.6 ± 1.2 mg/mL (*n* = 19) with no difference between diet groups. Exposure to BPS slightly decreased protein concentration in the overfed females exclusively (69.3 ± 1.0 mg/mL vs. 64.8 ± 1.3 mg/mL; *p*-value (t-test) = 0.04). In the following, the same amount of proteins per sample was used for gel migration and quantitative proteomic analyses.

The quality and homogeneity of samples were checked after the migration of 10 µg proteins on a 10% Mini-PROTEAN® TGX™ precast polyacrylamide gel (Bio Rad, Hercules, California, United-States) 10 min at 80 V and 30 min at 180 V followed by a PageBlue™ staining (ThermoFisher, Waltham, Massachusetts, United-States) (see Figure S1).

### In-gel protein digestion and nano-liquid chromatography coupled with tandem mass spectrometry (nanoLC-MS/MS)

For proteomic analysis, an aliquot of each pool was taken and diluted 1:10 in H_2_0 in order to reach a minimum volume of 20 µL. The rest of the pool, at least 50 µL, was used for steroidome analysis [14]. Then, 50 µg of protein from each sample was migrated on a 10% Mini-PROTEAN® TGX™ precast polyacrylamide gel for 10 min at 80 V. The gel was stained with PageBlue™, then each lane was cut horizontally in four slices for quantitative proteomic analysis. Gel pieces were washed in a water and acetonitrile solution (1:1, 5 min) followed by 100% acetonitrile (10 min). Reduction and cysteine alkylation was performed by successive incubation with 10 mM dithiothreitol in 50 mM NH4HCO3 (30 min, 56 °C), then 55 mM iodoacetamide in 50 mM NH_4_HCO_3_ (20 min at room temperature in the dark). Pieces were then incubated with 50 mM NH_4_HCO_3_ and acetonitrile (1:1, 10 min) followed by acetonitrile (15 min). Proteolytic digestion was carried out overnight using 25 mM NH4HCO3 with 12.5 ng/μl trypsin (Sequencing grade, Roche diagnostics, Paris, France). The resulting peptides were extracted by conducting an incubation in 5% formic acid and sonication, followed by incubation in acetonitrile and 1% formic acid (1:1, 10 min), and a final incubation with acetonitrile for 5 min. These two peptide extractions were pooled and dried using a SPD1010 Speedvac system (Thermosavant, Thermofisher Scientific, Bremen, Germany). The resulting peptide mixture was desalted and enriched with SPIN Columns C18 (Millipore) and dried again before being analyzed by nanoLC-MS/MS. All experiments were performed on a dual linear ion trap Fourier Transform Mass Spectrometer (FT-MS) LTQ Orbitrap Velos Pro (Thermo Fisher Scientific, Bremen, Germany) coupled to an Ultimate® 3000 RSLC Ultra High Pressure Liquid Chromatographer (Thermo Fisher Scientific, Bremen, Germany) controlled by Chromeleon Software (version 6.80 SR13). Samples were desalted and concentrated for 10 min at 5 µL/min on an LC Packings trap column (Acclaim PepMap 100 C18, 75-µm inner diameter x 2 cm long, 3 µm particles, 100 Å pores). The peptide separation was conducted using a LC Packings nano-column (Acclaim PepMap C18, 75 µm inner diameter × 50-cm long, 2-µm particles, 100 Å pores) at 300 nL/min by applying a gradient that consisted of 2–45% B during 90 min. Mobile phases consisted of (A) 0.1% formic acid, 97.9% water, 2% acetonitrile (v/v/v), and (B) 0.1% formic acid, 19.9% water, 80% acetonitrile (v/v/v). Data were acquired using the Xcalibur version 3.0.63 software (Thermo Fisher Scientific, San Jose, CA) in positive data-dependent mode in the 300–1800 m/z mass range. Resolution in the Orbitrap was set at R = 60,000. The 20 most intense peptide ions with charge states ≥ 2 were sequentially isolated (isolation width 2 m/z, 1 microscan), and fragmented in the high pressure linear ion trap using CID (collision induced dissociation) mode (collision energy 35%, activation time 10 ms, Qz 0.25). Dynamic exclusion was activated for 30 s with a repeat count of 1. A polydimethylcyclosiloxane (m/z, 445.1200025, (Si(CH3)2O)6) ion was used for internal recalibration of the mass spectra MS/MS.

### Protein identification, validation and quantification

Ion searches were performed using the Mascot search engine version 2.7.0.1 (Matrix Science, London, UK) with the NCBIprot_Mammals database (2021/07) and the Proteome Discoverer 2.5 software (ThermoFisher Scientific, Bremen, Germany). The search parameters included trypsin as a protease, with two allowed missed cleavages, and carbamidomethylcysteine, methionine oxidation, and acetylation of N-term protein as variable modifications. The tolerance of the ions was set to 5 ppm for parent, and 0.8 Da for fragment ion matches. The Mascot results obtained from the target and decoy database searches were subjected to Scaffold Q + S v5.1.1 and Scaffold Quant v5.0.3 software (Proteome Software, Portland, USA) using the protein cluster analysis option (assemblage of proteins into clusters based on shared peptide evidence). Peptide and protein identifications were accepted if they could be established at greater than 95% probability, as specified by the Peptide Prophet algorithm [53] and the Protein Prophet algorithm [54], respectively. Protein identifications were accepted if they contained at least two identified peptides. In addition to *Ovis aries*, the database of 18 herbivorous species were considered for peptide identification: *Bison bison bison, Bos indicus, Bos indicus x Bos taurus, Bos javanicus, Bos taurus, Bubalus bubalis, Camelus bactrianus, Camelus dromedaries, Camelus ferus, Capra hircus, Cervus elaphus hippelaphus, Equus asinus, Equus caballus, Equus przewalskii, Muntiacus muntjac, Muntiacus reevesi, Odocoileus virginianus texanus,* and *Vicugna pacos*. Finally, the database of *Homo sapiens,* which is much better annotated than the previous ones, was also considered for protein identification. The abundance of proteins was assessed by label-free quantification and the normalized weighted spectral (NWS) method, in which each peptide is assigned to a weight according to whether it is shared or not among proteins, was used for protein quantification [55].

### Prediction of secretory pathways

The prediction of conventional (presence of a signal peptide) and unconventional secretory pathways of identified proteins was performed as previously described [6]. Briefly, FASTA sequences of all identified proteins were retrieved from Uniprot ID mapping online tool, and were used as inputs into the Outcyte online tool (version 1.0; http://www.outcyte.com/) [56] and SignalP in Eukarya organism (version 6.0; https://services.healthtech.dtu.dk/services/SignalP-6.0/) [57]. Prediction of secretion by oviduct EVs was performed by comparing the list of genes of all identified proteins with the ones previously described in bovine [31] and human [32] oviduct EVs.

### Statistical analysis of quantified proteins

Statistical analysis was performed using the Rstudio software (version 2023.06). Only proteins quantified with a mean quantitative value of at least 2 NWS in one condition were considered for statistical analysis. PCA on all quantified proteins was carried out using the FactoMineR package. The effects of the diet and exposure to bisphenol S on protein abundance were analyzed by ANOVA (*p*-value ≤ 0.05) and Student’s t-tests (*p*-value ≤ 0.05 and fold-change ratio ≥1.5 ). Hierarchical clustering and heatmap were performed on differentially abundant proteins (DAPs) using the gplots package. Data are presented as means ± SEM.

### Functional enrichment analysis of differentially abundant proteins

The lists of orthologous genes in *Homo sapiens* corresponding to the DAPs were used as inputs for functional analysis. The GO enrichment analysis of DAPs was first performed using the Metascape online tool [58]. Overrepresented BP GO terms and Reactome pathways with a *p*-value ≤ 0.05 were considered as significant. The BP GO terms, Kyoto Encyclopedia of Genes and Genomes (KEGG), and Reactome pathways were further analyzed using the Database for Annotation, Visualization and Integrated Discovery (DAVID). Overrepresented GO terms and pathways with a *p*-value ≤ 0.05 were considered as significant [59].

### Supplementary Information

Additional file 1: Figure S1

Additional file 2: Table S1

Additional file 3: Table S2

Additional file 4: Table S3

## Data Availability

The datasets generated during the current study are available in the ProteomeXchange Consortium [60] via the PRIDE [61] partner repository with the dataset identifier PXD044858.

## Abbreviations

Bisphenol S: BPS
BCS: Body condition score
BW: Body weight
DAPs: Differentially abundant proteins
DFR: Daily food requirements
EVs: Extracellular vesicles
GO BP: Gene ontology biological process
NanoLC-MS/MS: Nano-liquid chromatography coupled with tandem mass spectrometry
NEFA: Non-esterified fatty acids
NWS: Normalized weighted spectra
OF0: Overfed diet and non-exposed to bisphenol S
OF50: Overfed diet and exposed to 50 µg/kg/day of bisphenol S
OF50: Underfed diet and non-exposed to bisphenol S
UF50: Underfed diet and exposed to 50 µg/kg/day of bisphenol S
