# Correction: Oral exposure to bisphenol S is associated with alterations in the oviduct proteome of an ovine model, with aggravated effects in overfed females

**DOI:** 10.1186/s12864-024-10541-6

**Published:** 2024-07-02

**Authors:** Coline Mahé, Marie-Emilie Lebachelier de la Riviere, Olivier Lasserre, Guillaume Tsikis, Daniel Tomas, Valérie Labas, Sébastien Elis, Marie Saint-Dizier

**Affiliations:** 1 INRAE, CNRS, Université de Tours, PRC, Nouzilly, 37380 France; 2 INRAE, PAO, Nouzilly, 37380 France; 3 INRAE, Université de Tours, CHU de Tours, Plateforme de Phénotypage Par Imagerie in/eX Vivo de L’ANImal À La Molécule (PIXANIM), Nouzilly, 37380 France

**Correction** **:** **BMC Genomics 25, 589 (2024)**

**https://doi.org/10.1186/s12864-024-10510-z**

Following publication of the original article [1] it was reported that the following text in the “Profiling of the oviduct fluid proteome according to the exposure to BPS and diet” section should be corrected:

**Original text:** The GO analysis of proteins in cluster 1 evidenced an enrichment in 19 biological processes (BP) and pathways, including six related to metabolism, three related to the immune system, three to the cell response to stress, and three to cell movement among the most significant ones (p-value < 0.01) (Table 1). Analysis of proteins in cluster 2 showed an enrichment in 16 BP and pathways, including two related to intracellular trafficking, five to metabolism, three to immune system, and one related to the negative regulation of reproductive process (Table 2).

**Corrected text: **The GO analysis of proteins in cluster 1 evidenced an enrichment in 16 BP and pathways, including two related to intracellular trafficking, five to metabolism, three to immune system, and one related to the negative regulation of reproductive process (Table 1). Analysis of proteins in cluster 2 showed an enrichment in 18 biological processes (BP) and pathways, including six related to metabolism, two related to the immune system, three to the cell response to stress, and three to cell movement among the most significant ones (p-value < 0.01) (Table 2).

The original article has been updated.
