# Supplementary material for: Oral exposure to bisphenol S is associated with alterations in the oviduct proteome of an ovine model, with aggravated effects in overfed females
Source: BMC Genomics. 2024 Jun 12;25:589. doi: 10.1186/s12864-024-10510-z (PMC11167748; doi:10.1186/s12864-024-10510-z)
Supplement: Supplementary file 1 — Additional file 1: Figure S1 [file 12864_2024_10510_MOESM1_ESM.pdf]

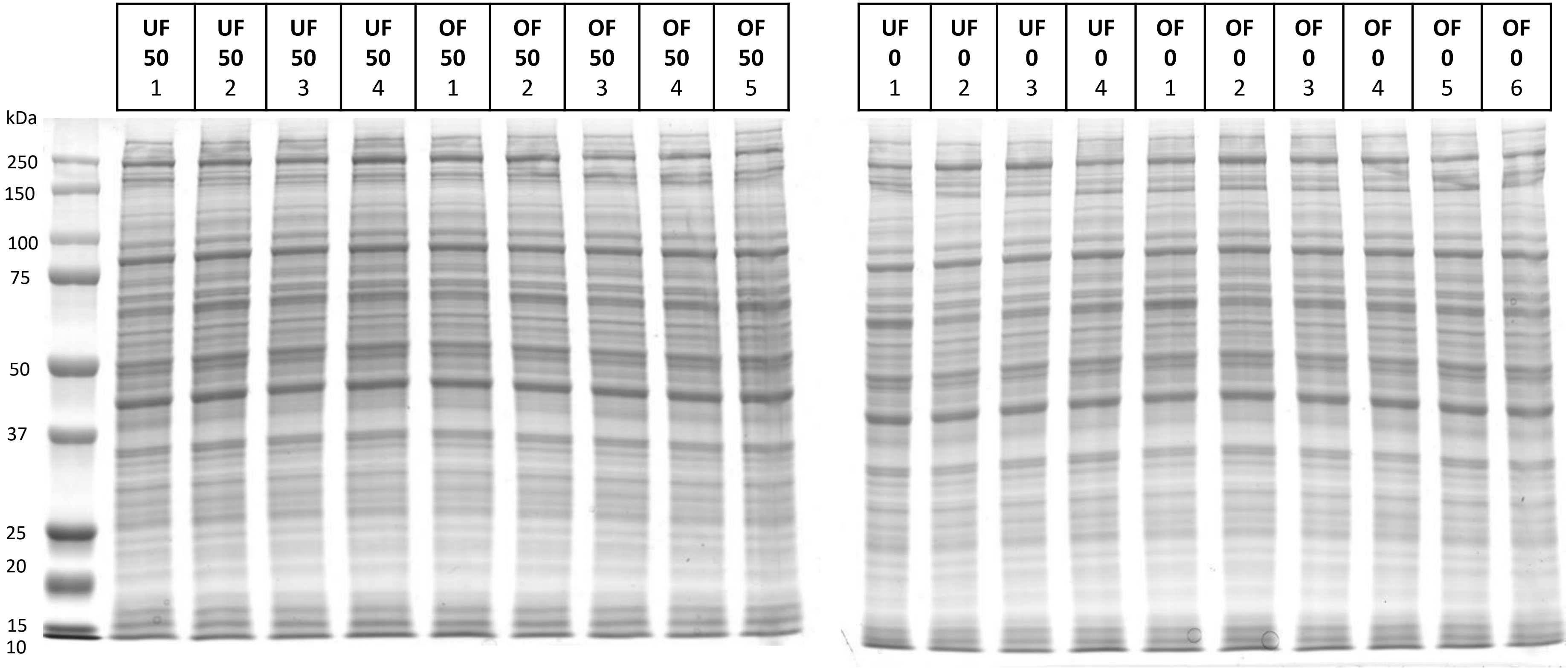

Additional file 1 : Figure S1: Protein profile of the 19 samples of ovine oviduct fluid
10 µg of oviduct fluid were migrated on a 10% SDS-page pre-cast gel and stained with Coomassie blue
